# The Older Methods of Filling Teeth

**Published:** 1903-06

**Authors:** C. N. Peirce


### Domestic Correspondence.

### THE OLDER METHODS OF FILLING TEETH.

To the Editor:

Sir,—I have just read the article in the April number on the progress of tooth-filling in fifty years with a great deal of interest. It takes me back to the first operation I saw Dr. Arthur perform, and then to my own efforts the following day in filling with annealed gold. The operations that were performed then and in subsequent years were many, and probably there were not three years of more unsatisfactory practice in tooth-filling ever performed than resulted from the efforts to use annealed foil without appreciating the essentials and without proper instruments for the performance of the work. Finely serrated instruments, electric and hand mallets, and small pellets of gold were all the result of the new method, and to obtain these and to learn how to manipulate with them required more than a day's experience. The new process involved more than ordinary skill. Principles were involved with which the operator had to make himself familiar, and not until this was done was he in a condition to perform successful or preservative work. The ignorance of the operator was certainly the reason that so much unsatisfactory work was done. Many consecutive hours did I watch my preceptor, F. M. Dixon, perform contour fillings with soft foil, but they were made with deeply serrated instruments and hand pressure. I do not think he ever had a mallet in his office. In durability and comfort his work was not to be excelled. This much could not be said of Dr. Webb's operations. Many of his two and three days' work were failures, because the endurance of the periosteum was ignored. This has been an error on the part of many beautiful adhesive gold-foil operators. Teeth and fillings have been sacrificed after a few months of discomfort, where the patient's endurance in the operation was not the only tax they suffered.

I am glad that this method of practice has given way to not only less exhaustive treatment, but to one more in harmony with the possibilities of tooth-structure.

C. N. Peirce.

